# Prevention of Adverse Events in Endoscopic Ultrasound‐Guided Biliary Drainage

**DOI:** 10.1002/deo2.70145

**Published:** 2025-05-22

**Authors:** Hirotoshi Ishiwatari, Hiroki Sakamoto, Takuya Doi, Masahiro Yamamura

**Affiliations:** ^1^ Division of Endoscopy Shizuoka Cancer Center Shizuoka Japan

**Keywords:** adverse events, biliary drainage, choledochoduodenostomy, endoscopic ultrasound, hepaticogastrostomy

## Abstract

Endoscopic ultrasound‐guided biliary drainage (EUS‐BD) is used when biliary drainage using endoscopic retrograde cholangiopancreatography fails. Recently, it has been adopted as a primary biliary drainage method, and its indications have expanded. Since EUS‐BD can cause adverse events (AEs), such as bile leakage and stent migration, which do not occur in endoscopic retrograde cholangiopancreatography, endoscopists need to be well‐versed in its management and preventive techniques. EUS‐BD includes several procedures, such as EUS‐guided choledochoduodenostomy (EUS‐CDS), EUS‐guided hepaticogastrostomy (EUS‐HGS), EUS‐guided antegrade stenting (EUS‐AS), and EUS‐guided rendezvous (EUS‐RV). A recent meta‐analysis reported that the overall AE rate of EUS‐BD was 13.7% (EUS‐CDS, 11.9%; EUS‐HGS, 15.5%; EUS‐AS, 9.9%; and EUS‐RV, 8.8%). Among various EUS‐BD techniques, EUS‐CDS and EUS‐HGS are the most frequently reported. Tubular self‐expandable metal stents have been traditionally used in EUS‐CDS; however, lumen‐apposing metal stents have recently gained popularity. A systematic review showed that the rates of early AEs were similar between self‐expandable metal stents and lumen‐apposing metal stents; however, stent maldeployment was more problematic with lumen‐apposing metal stents. Although tubular self‐expandable metal stents are used in EUS‐HGS, stent maldeployment remains a serious issue, and available devices and technical tips for preventing this AE should be well understood. Furthermore, AEs, such as sepsis, cholangitis, and bleeding, can occur, and strategies to mitigate these risks are essential. In this narrative review, we discussed AEs related to EUS‐BD with a focus on management options and strategies for prevention.

## 1 Introduction

A standard biliary drainage procedure involves endoscopic biliary stenting using endoscopic retrograde cholangiopancreatography (ERCP) [1, 2]. However, ERCP is not feasible in patients with duodenal obstruction, surgically altered anatomy, or failed biliary cannulation, and percutaneous transhepatic biliary drainage (PTBD) has long been performed to manage these challenging situations. However, since endoscopic ultrasound‐guided biliary drainage (EUS‐BD) was first introduced in the early 2000s, it has largely replaced PTBD and has been adopted in many high‐volume centers [3, 4, 5, 6, 7]. EUS‐BD includes several procedures such as EUS‐guided choledochoduodenostomy (EUS‐CDS), EUS‐guided hepaticogastrostomy (EUS‐HGS), EUS‐guided antegrade stenting (EUS‐AS), and EUS‐guided rendezvous (EUS‐RV) [3, 8–24]. Recently, the combination of EUS‐HGS and EUS‐AS has been reported and is referred to as EUS‐HGAS [14, 15, 16, 17, 18]. Unlike ERCP, EUS‐BD creates a fistula to connect two organs, such as the bile duct and the gastrointestinal tract, which can lead to adverse events (AEs) that never occur with ERCP [2, 3, 4, 5]. Therefore, endoscopists must be well‐versed not only in managing these AEs but also in preventing them. In this narrative review, we discussed the AEs associated with EUS‐BD procedures, as well as management options and strategies for prevention.

## 2 AEs of EUS‐BD

**FIGURE 1:**
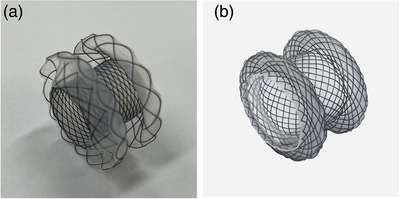
Lumen‐apposing metal stent: (a) Hot‐AXIOS System (Boston Scientific). (b) Niti‐S HOT SPAXUS stent (Taewoong Medical).

A meta‐analysis conducted in 2023 included 7887 patients from 155 studies and evaluated the AEs associated with EUS‐BD. Overall AE rate was 13.7% (EUS‐CDS, 11.9%; EUS‐HGS, 15.5%; EUS‐AS, 9.9%; and EUS‐RV, 8.8%) [9]. Additionally, the overall major AE and mortality rates were 0.6% and 0.1%, respectively. These are consistent with the rates reported in the 2019 guideline, which indicated AE rates of 13.9%, 18.2%, and 12.4% for EUS‐CDS, EUS‐HGS, and EUS‐RV, respectively [3]. Among these procedures, EUS‐CDS and HGS have been reported most frequently. Two randomized controlled trials comparing these two techniques found no significant difference in AE rates (12.5% vs. 20% [*p* = 0.70] and 17.4% vs. 25.0% [*p* = 0.52]) [25, 26]. Furthermore, a meta‐analysis that included both prospective and retrospective studies showed no significant difference in AE rates between EUS‐CDS and HGS (odds ratio [OR] = 0.97; 95% confidence interval [CI] = 0.60–1.56) [27]. Previously, tubular‐covered self‐expandable metal stents (SEMS) were used for EUS‐CDS; however, they have been recently replaced by lumen‐apposing metal stents (LAMS; Figure 1) [25, 28, 29]. When using an SEMS, multiple steps, such as guidewire insertion and fistula dilation, are required before stent placement. In contrast, LAMS allows these steps to be performed in a single procedure, which is expected to reduce the incidence of AEs such as bile leakage and bleeding. Although no randomized controlled trials comparing the two devices have been conducted to date, a meta‐analysis comparing SEMS and LAMS showed no significant difference between the two in terms of overall AE rates (SEMS: 18.3% [95% CI: 14.3%–23.0%] vs. LAMS: 17.1% [95% CI: 12.5%–22.8%]), or severe AE rates (SEMS: 2.6% [95% CI: 1.5%–4.4%] vs. LAMS: 1.4% [95% CI: 0.5%–3.6%]) [30]. EUS‐HGAS has been reported more recently, with overall AE rates between 10% and 18.5% [14, 15, 18]. A multicenter, prospective study including 49 patients reported an overall AE rate of 10.2% [14]. Representative AEs include bile leak, bleeding, stent migration, cholangitis, and sepsis with individual AE rates reported to be between 0% and 15% [3, 9, 31].

### 2.1 Bile Leakage (Peritonitis)

**FIGURE 2:**
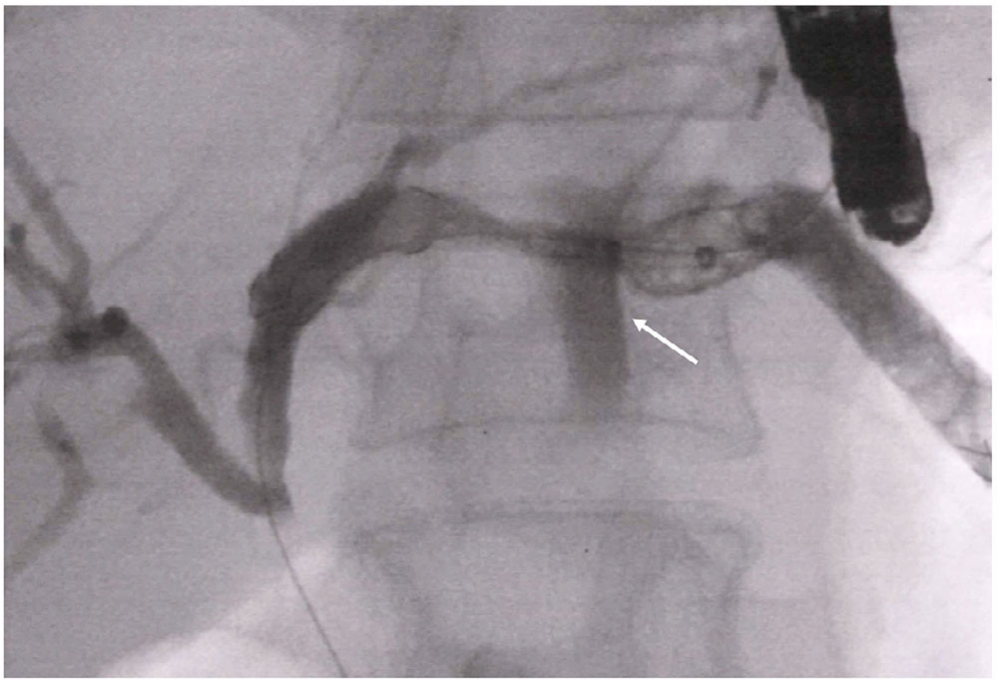
Bile leakage due to damage to the cover membrane of an HGS stent. This case involved EUS‐HGS with a partially covered self‐expandable metal stent. The day after the procedure, the patient complained of abdominal pain and computed tomography revealed fluid collection around the stomach and spleen. Balloon‐occluded cholangiography demonstrated contrast leakage from the HGS stent between the stomach and the liver (→), necessitating the placement of an additional covered metal stent inside the HGS stent to seal the leak. EUS‐HGS, endoscopic ultrasound‐guided hepaticogastrostomy.

A meta‐analysis evaluating stent placement by ERCP and EUS‐BD reported that the incidence of bile leakage was 2.2% among patients who underwent EUS‐BD [32]. However, the diagnosis of bile leakage or peritonitis can be challenging. Although to evaluate AEs, most studies used the Lexicon criteria, AGREE classification, or Common Terminology Criteria for Adverse Events; however, these criteria do not define bile leakage or peritonitis. Furthermore, some studies proposed their own definitions for the evaluation [33, 34]. A prospective study defined peritonitis as the presence of clinical symptoms of peritoneal inflammation along with evidence of corresponding fluid collection on computed tomography [35]. In contrast, another retrospective multicenter study defined it as the presence of abdominal rebound tenderness with new‐onset abdominal pain [18]. Therefore, cases of mild peritonitis may have been overlooked, potentially leading to an underestimation of the reported incidence rate. If this AE is not managed properly, it can be fatal, especially in cases where surgery is contraindicated due to severe comorbidities [36]. These AEs occur not only during the procedure but also after stent placement. SEMS is considered less likely to cause bile leaks because of their self‐expandable properties. However, bile leakage can still occur, albeit rarely, owing to damage to the cover membrane of the stent (Figure 2). Additionally, when using SEMS or plastic stents (PS), stent occlusion or increased intragastric pressure before fistula maturation can lead to bile leakage and peritonitis. These AEs have been reported to be treated by performing AS for the biliary obstruction site or by placing a duodenal stent [37, 38].

### 2.2 Bleeding

**FIGURE 3:**
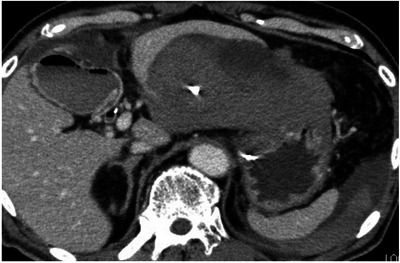
Bleeding after EUS‐HGS using a plastic stent. Computed tomography performed one day after EUS‐HGS showed a huge hematoma between the liver and stomach. This probably resulted from multiple punctures due to targeting of the small bile ducts. EUS‐HGS, endoscopic ultrasound‐guided hepaticogastrostomy.

Bleeding occurs in 0.5%–3.7% of patients after EUS‐BD [3]. The main cause is accidental puncture of blood vessels during bile duct puncture, whereas fistula dilation using a cautery dilator or a balloon catheter may also lead to bleeding (Figure 3). These events result in hemorrhage inside the digestive tract or hematoma outside the digestive tract and can be fatal [36, 39]. In cases where bleeding occurs inside the digestive tract, endoscopic hemostasis by epinephrine injection into the gastroduodenal wall can be a treatment option. Theoretically, PS may carry a higher risk of bleeding compared to SEMS and LAMS, as the expansion of SEMS and LAMS can promote hemostasis through pressure. However, this assumption is not supported by evidence. The reported rate of bleeding after PS placement is 3.5%–5.6% [40, 41]. In cases of LAMS, dilation may be useful in stopping bleeding by increasing the tamponade effect. However, it is important to note that LAMS dilation has been reported to induce bleeding in EUS‐guided gastroenterostomy using LAMS, which may also occur in EUS‐CDS using LAMS [42, 43, 44]. Furthermore, pseudoaneurysm formation around a stent as a late AE can cause bleeding and may require transcatheter embolization in some cases [45, 46]. The use of antithrombotic agents raises concerns about the risk of bleeding. However, a single‐center retrospective study showed that the risk did not increase in cases where these drugs were temporarily suspended in accordance with guidelines [47, 48].

### 2.3 Stent Migration

Stent migration can occur in 1.6‐5.8% of EUS‐HGS and CDS cases [3, 49]. It can occur during the procedure because of stent maldeployment. Moreover, a stent can be dislodged after placement due to stent shortening and bowel movements. This AE can lead to bile leakage and peritonitis, which requires urgent management. Several rescue methods have been reported for migrating the HGS. Cases in which a migrated stent was punctured under EUS guidance, followed by the placement of an additional SEMS using a stent‐in‐stent technique have been documented [50, 51, 52, 53]. Other cases involved removing the migrated stent using natural orifice transluminal endoscopic surgery [54]. Additionally, when a stent has not completely migrated into the abdominal cavity, two methods have been documented: (1) securing the HGS SEMS by skewering the SEMS using a PS and (2) using a detachable snare [55, 56]. In EUS‐CDS, stent migration appears to be more problematic when using LAMS compared with SEMS. A systematic review of EUS‐CDS using LAMS, including 1081 patients from 25 studies, reported that migration occurred in 5.8% of cases, with 86.8% of these being managed endoscopically [49]. Furthermore, a retrospective, single‐center study comparing the two devices found that stent maldeployment and dislodgement occurred in 5.4% and 5.4% of cases after LAMS placement, respectively, while no such events were reported after SEMS placement [57]. Furthermore, a multicenter, retrospective study analyzed 100 technical failure cases from 23 hospitals in Europe [58]. Multivariable analysis identified the following as significant risk factors for technical failure: common bile duct diameter ≤15 mm, duodenal stenosis, wired technique, and low operator experience (≤10 LAMS). The most common reason for failure was malpositioning of the distal flange of the LAMS in the bile duct (41%), followed by malpositioning of the proximal flange in the digestive tract (26%). Endoscopic treatment was successful in 77% of the failed cases. However, a 30‐day mortality rate after the failure was 12%. The risk of LAMS migration is higher than that of tubular SEMS, likely due to the short length of LAMS, which makes positioning of LAMS more challenging [42, 57]. As for the salvage method for LAMS migration, when a guidewire is placed in the bile duct, one option is to insert a SEMS inside the LAMS to bridge the bile duct and the duodenum [59]. If a guidewire does not advance into the liver but enters the duodenum, placing an SEMS via the duodenal major papilla using the rendezvous technique can be considered [60]. When a guidewire cannot be placed in the bile duct, a repeat EUS‐CDS attempt may be necessary [59]. If this is not feasible, ERCP or PTBD would be required.

### 2.4 Pancreatitis

If a stent is placed across the major duodenal papilla when performing EUS‐AS or EUS‐HGAS, pancreatitis can occur, with a reported incidence rate of 0%–13% of cases [11, 13–16, 18, 61, 62]. Although no mortality has been reported, this may be due to the limited number of cases. This technique was originally used for PTBD and has since been applied in EUS‐AS. Mortality after severe pancreatitis has been reported in cases of stent placement via PTBD [63]. Therefore, caution should be exercised when performing EUS‐AS.

### 2.5 Rare AEs

The following AEs are rare; however, they should be recognized as important potential AEs.

#### 2.5.1 Guidewire Malposition

When inserting a guidewire after a bile duct puncture, the guidewire can sometimes enter the space adjacent to the bile duct while appearing to be inside it [64]. If a stent is placed without recognizing the guidewire malposition, bile drainage may not be achieved properly. This AE is particularly likely when targeting the intrahepatic bile duct in segments 2 or 3 because these ducts are typically small, making it difficult to clearly visualize the guidewire within the bile duct using EUS.

#### 2.5.2 Hepatic Abscess and Biloma

An HGS stent, whether fully or partially covered, can potentially obstruct the peripheral bile duct, leading to the formation of a hepatic abscess or biloma. If the collection develops near the digestive tract, endoscopic drainage with stent placement may be an option [65, 66]. Otherwise, percutaneous drainage is required.

#### 2.5.3 HGS Stent Migration into the Esophagus and Mediastinitis

A HGS stent can migrate into the esophagus and cause vomiting. In most cases, it can be repositioned endoscopically by pushing it back into the stomach or by placing an additional stent inside the HGS stent. However, a case requiring surgical removal of the HGS stent due to repeated migration into the esophagus has been reported [67]. Additionally, a migrated HGS stent into the esophagus causing esophageal perforation and mediastinitis has also been documented [68]. This AE is more likely to occur when a long stent (≥10 cm) is used for HGS.

#### 2.5.4 Portal Vein Puncture

Accidental portal vein puncture can occur during EUS‐CDS when the extrahepatic bile duct is being targeted. Cases involving portal vein puncture using a 19‐gauge needle, followed by catheter insertion over the guidewire or using the cautery tip of LAMS have been reported [70, 71]. In the former case, the guidewire was removed, and EUS‐CDS using a SEMS was successfully performed without AEs [70]. In the latter case, massive bleeding occurred after successful LAMS placement in the bile duct, and then SEMS was placed via ERCP using the rendezvous technique to seal the portal vein puncture site [71]. If a stent is inadvertently placed in the portal vein, it can be fatal.

## 3 How to Prevent AEs

### 3.1 Careful Observation (for All EUS‐BD)

It is crucial to carefully observe both endoscopic and EUS views to avoid puncturing blood vessels and the intestines located between the echoendoscope and the target organ. Additionally, color Doppler EUS should be used to identify intervening blood vessels. However, digestive tract compression by echoendoscopy may obscure blood vessels on EUS images. Therefore, it is essential to obtain EUS images while alternately applying and releasing pressure on the digestive tract. Contrast‐enhanced computed tomography is also valuable for identifying large blood vessels around an expected puncture site before performing EUS‐BD.

### 3.2 Avoid Puncturing Through the Mediastinum (for EUS‐HGS)

**FIGURE 4:**
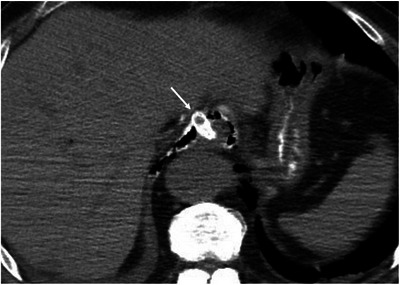
Mediastinitis after EUS‐HGS. During EUS‐HGS, the intrahepatic bile duct in segment 2 was punctured, resulting in the placement of a covered metal stent from the esophagus. Three days after the procedure, the patient developed a high fever, and computed tomography revealed air bubbles around the HGS stent in the mediastinum (→), suggesting mediastinitis. EUS‐HGS, endoscopic ultrasound‐guided hepaticogastrostomy.

For EUS‐HGS, puncturing via the mediastinum increases the risk of mediastinitis, especially when targeting the intrahepatic bile duct in segment 2 (Figure 4) [72]. To mitigate this risk, it is important to identify the location of the diaphragm on EUS images and ensure that puncture occurs below it. However, visualizing the diaphragm on EUS images can be challenging. In such cases, attaching a hemostatic clip at the esophagogastric junction and visualizing it under fluoroscopy can help guide the puncture from the stomach. Although puncturing from the esophagus does not always mean that the needle passes through the mediastinum, when the EUS images show an unclear diaphragm and there is uncertainty about whether the needle might pass through the mediastinum, it is safer to puncture below the esophagogastric junction to perform the procedure safely.

### 3.3 Metallic Stent (for EUS‐CDS and HGS)

Although there have been a few studies comparing SEMS and PS, an international, multicenter, retrospective study conducted in 2016 evaluated 121 patients who underwent EUS‐BD (CDS: 60 and HGS: 61) and found a significantly higher overall AE rate in PS compared with SEMS (42.3% vs. 13.1%, OR: 4.99 [95% CI: 1.5–16.51], *p* = 0.01) [72]. Additionally, PS was identified as an independent predictor of AE in multivariable analysis (OR: 4.95 [95% CI: 1.41–17.38], *p* = 0.01) [72]. Furthermore, another international, multicenter, retrospective study published in 2014 showed no significant difference in the overall AE rate between SEMS and PS. However, a significantly higher incidence of cholangitis was found in patients with PS (11% vs. 3%, *p* = 0.02), although there was no difference in bile leakage between the two groups (9.3% vs. 9.2%; *p* = 0.97) [73]. Based on these results, guidelines recommend SEMS placement when a patient's clinical condition allows for its use [3, 5]. However, recent retrospective studies have reported a similar AE rate between SEMS and PS [74, 75]. In a single‐center, retrospective study, HGS and CDS cases were compared after balancing the two groups using a propensity score matching method, with no significant difference in AE rates between SEMS and PS (20% for SEMS vs. 16% for PS, *p* = 0.5) [74]. The occurrence of AEs with PS may have reduced due to the improvement in devices and techniques.

### 3.4 Stent Configuration to Prevent Stent Migration (for EUS‐CDS and HGS)

**FIGURE 5:**
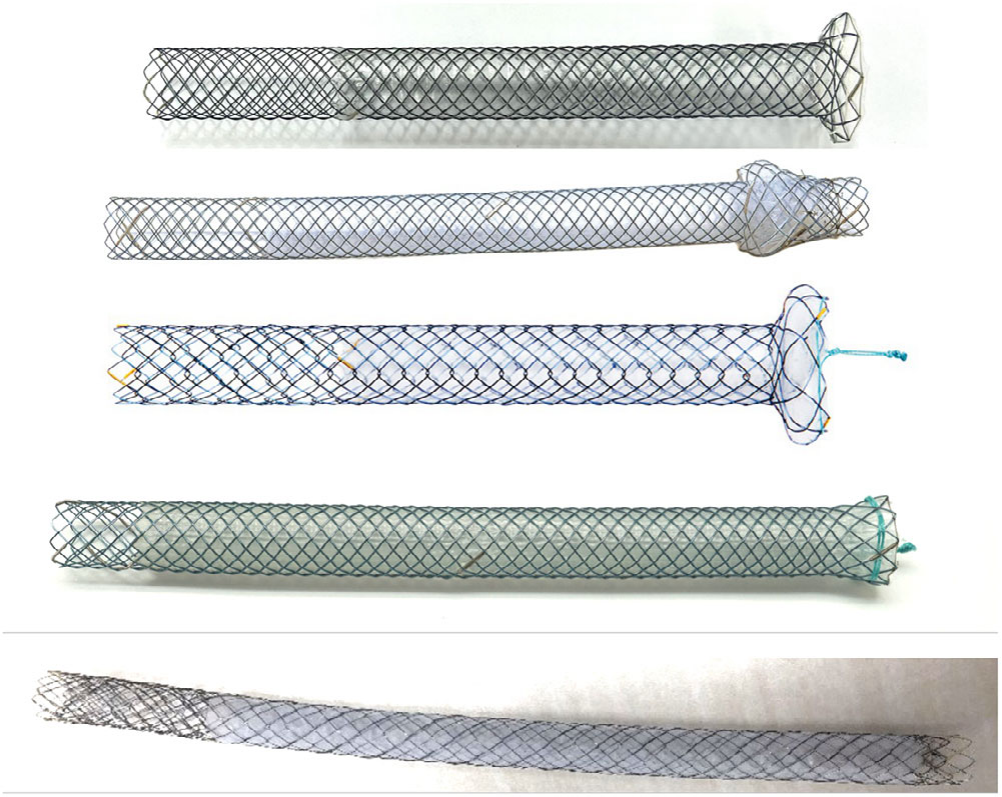
Covered self‐expandable metal stent for EUS‐HGS. From the top, GIOBOR (Taewoong Medical), Niti‐S Spring Stopper (Taewoong Medical), HANARO STENT (BPD; M.I.Tech), Niti‐S S‐type stent (Taewoong Medical), EGIS biliary (S&G Biotech Inc.). EUS‐HGS, endoscopic ultrasound‐guided hepaticogastrostomy.

**FIGURE 6:**
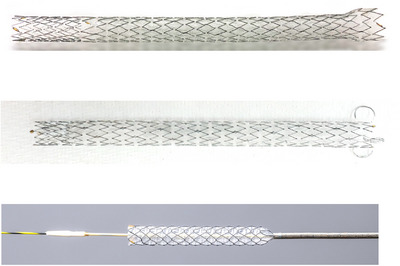
Covered self‐expandable metal stent for EUS‐HGS with a deliver system <7.5 Fr. BileRush Selective (Piolax), ZEOSTENT HG (Zeon Medical), and HANAROSTENT Benefit (M.I.Tech Co., Ltd.). EUS‐HGS, endoscopic ultrasound‐guided hepaticogastrostomy.

A tubular‐covered SEMS with anti‐migratory properties would be optimal, especially for EUS‐HGS, to prevent stent migration caused by stent shortening and bowel movement [76, 77, 78]. Various types of SEMS are used for EUS‐HGS; however, their availability varies by country (Figure 5). A single‐center, retrospective study evaluating the Giobor stent with an 8.5 Fr delivery system (Taewoong Medical, Korea) reported stent migration in 2 of 41 patients who underwent EUS‐HGS, though these AEs were successfully managed endoscopically [76]. Additionally, a single‐center, prospective study assessed a partially covered SEMS with proximal and distal anchoring flaps, featuring an 8 Fr delivery system (Standard Sci Tech Inc., Korea); they found no cases of stent migration after 21 EUS‐HGS and 33 EUS‐CDS procedures [77]. When SEMS with anti‐migratory features are unavailable, a long SEMS (≥10 cm) can be an alternative. A multicenter, retrospective study reported that no cases of stent dislocation occurred in 110 EUS‐HGS using a long SEMS [79]. Although these SEMSs are of the braided type, laser‐cut SEMSs for EUS‐HGS have recently become available (Figure 6). A single‐center, retrospective study comparing these two types found that the distance between the hepatic parenchyma and stomach wall at days one and seven post‐EUS‐HGS was shorter in a laser‐cut SEMS group, suggesting that laser‐cut SEMS may reduce the risk of stent migration [80]. More recently, a newly designed, partially covered laser‐cut SEMS with an antimigration anchoring hook has been developed and evaluated in a multicenter, prospective study (Figure 6) [78]. This study included 38 EUS‐HGS cases and reported no instances of stent migration after the procedure. Furthermore, this SEMS features a thin, tapered tip with a 7.2 Fr delivery system, eliminating the need for tract dilation in 94.7% of the enrolled patients.

### 3.5 Slim Delivery System of Covered Metallic Stent (for EUS‐CDS and HGS)

The slim SEMS delivery system has the advantage of bypassing tract dilation, which may reduce the incidence of early AEs (Figure 6). A retrospective study revealed that the use of an SEMS with a delivery diameter of <7.5 Fr required tract dilation in 18% of EUS‐BD cases. In contrast, when using SEMS with a delivery diameter ≥7.5 Fr, tract dilation is required in 67% of cases (*p* < 0.001) [81]. Additionally, the early AE rate was significantly lower with SEMS having a delivery diameter of <7.5 Fr (12% vs. 35%, *p* = 0.01). A slimmer delivery system is advantageous when inserting SEMS not only for EUS‐CDS and HGS but also for EUS‐AS [82]. However, since there are concerns regarding the durability of both the stent itself and its covering membrane in slimmer covered SEMS ≤6 Fr. There is a trade‐off between delivery system diameter and SEMS characteristics, such as axial and radial forces and overall durability. Nevertheless, the ease of inserting an SEMS of ≤6 Fr can be beneficial in cases where inserting a conventional SEMS is challenging.

### 3.6 Intrascope Channel Release Technique (for EUS‐CDS and HGS)

**FIGURE 7:**
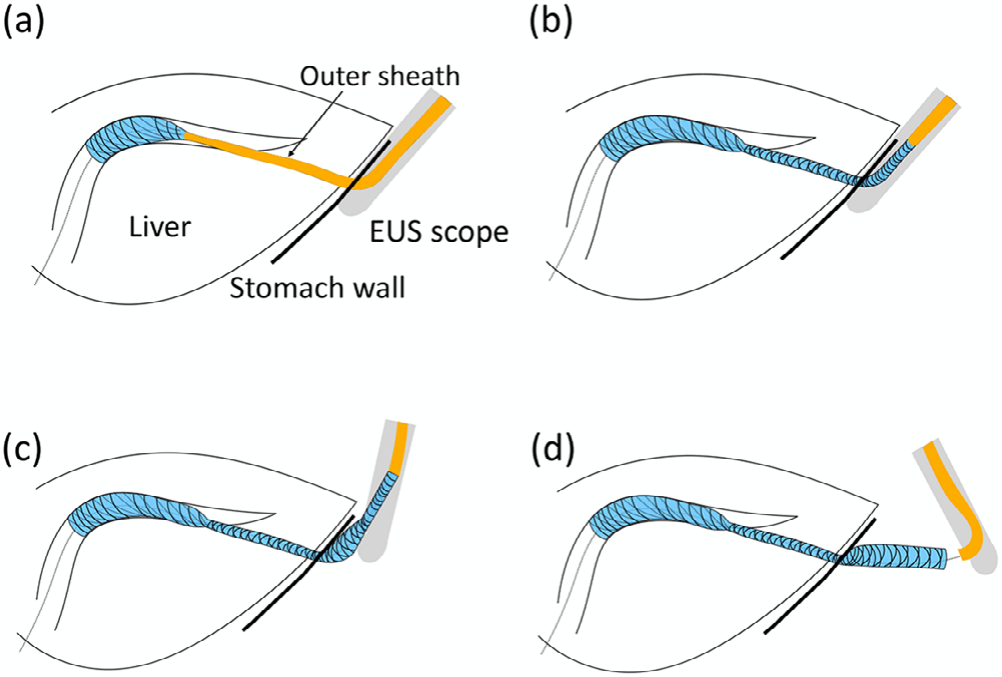
Intrascope channel release technique. (a) Open the distal part of the metal stent after inserting the stent delivery system over a guidewire. (b) Partially deploy the stent by retracting the outer sheath, then pull the outer sheath into the channel without moving the EUS scope under fluoroscopic guidance. (c) Visualize the body of the stent in the endoscopic view by pushing the outer sheath while slowly retracting the EUS scope. (d) Fully deploy the stent in the digestive tract by continuing to push the outer sheath. EUS, endoscopic ultrasound.

This technique safely deploys the proximal side of a stent in the digestive tract. Technically, after expanding the distal side of the stent under fluoroscopic or EUS guidance, the outer sheath of the delivery system is slowly withdrawn into the echoendoscope channel without retracting the echoendoscope itself (Figure 7). Next, the echoendoscope is moved slightly away from the digestive tract, and the stent body is visualized by advancing the stent delivery system. The system is continuously pushed to fully expand the proximal side of the stent. A single‐center, retrospective study of EUS‐HGS reported that no stent migration was observed in an intrascopic channel release group (0/21), whereas stent migration occurred in one patient in an extrascopic channel release group (1/20). However, no significant difference was found in early AE rates between the two groups [83]. Regardless of the type of stent used (SEMS or LAMS), the proximal end should theoretically be able to expand safely on the gastrointestinal side. Therefore, this technique is highly useful in preventing stent malpositioning.

### 3.7 Bile Aspiration (for EUS‐HGS)

Bile aspiration during EUS‐HGS may reduce the risk of early AEs. In cases where the bile duct is dilated owing to bile duct stones and biliary obstruction, the internal pressure of the bile duct is expected to be high. Therefore, during ERCP for obstructive cholangitis, it is crucial to aspirate bile once the ERCP catheter reaches the bile duct before injecting a contrast medium. Failure to do so may worsen cholangitis and lead to sepsis. Similar AEs may occur during EUS‐HGS. A single‐center, retrospective study of 96 EUS‐HGS cases found that aspiration of >10 mL of bile remained a significant factor in reducing early AEs in multivariable analysis [84]. The study indicated that bile aspiration of >10 mL contributed to lowering the incidence of early AEs, such as fever, abdominal pain, sepsis, and cholangitis [84]. In this study, an ERCP catheter was inserted into the bile duct after guidewire insertion, followed by bile aspiration through the catheter.

### 3.8 Double Guidewire Technique (for EUS‐HGS)

**FIGURE 8:**
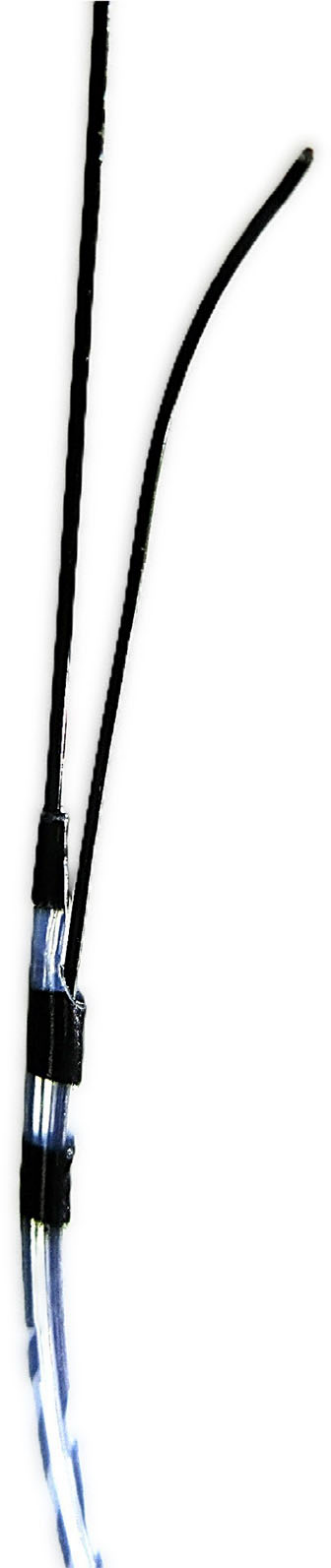
Uneven double‐lumen cannula (Piolax Medical Device). The catheter has two lumens; one exits from the tip of the catheter and accommodates a 0.025‐inch guidewire, whereas the other exits 5 mm behind the tip and allows the passage of a 0.035‐inch guidewire.

This technique involves using two guidewires, which are likely to facilitate subsequent fistula dilation and stent insertion during EUS‐HGS. A single‐center, retrospective study found that the technical success rate of EUS‐HGS using two guidewires was significantly higher than that using a single guidewire [75]. Furthermore, although the use of two guidewires was not identified as an independent factor influencing the occurrence of AEs in a multivariable analysis, additional fistula dilation was found to be a significant factor. Additionally, a multivariable analysis revealed that a guidewire angle at the insertion site >137° and intrahepatic bile duct diameter at the puncture site ≤3.0 mm were associated with the necessity of additional fistula dilation. The use of two guidewires can straighten the angle between the needle tract and bile duct, facilitating fistula dilation and potentially eliminating the need for additional fistula dilation. A double‐lumen catheter is useful for placing two guidewires (Figure 8) [85].

### 3.9 Long Needle Tract in the Hepatic Parenchyma (for EUS‐HGS)

Choosing a puncture site with a long needle tract in the liver may reduce the risk of bile leakage during EUS‐HGS. A single‐center, retrospective study evaluating risk factors for bile leakage during EUS‐HGS identified hepatic parenchyma length of the needle tract <2.5 cm as a significant factor associated with bile peritonitis [86]. Considering that bile leakage is more likely to occur when a guidewire is placed in the extrahepatic bile duct or gallbladder, where there is no hepatic parenchyma, this result seems plausible. A short needle tract in the hepatic parenchyma may increase the risk of bile leakage during EUS‐HGS. However, it is also true that multiple puncture sites are not always feasible. Therefore, if the needle tract in the hepatic parenchyma is short, other measures to reduce the risk of bile leakage, such as SEMS placement and bile aspiration, should be considered.

### 3.10 Push Deployment Technique (for EUS‐CDS using LAMS)

**FIGURE 9:**
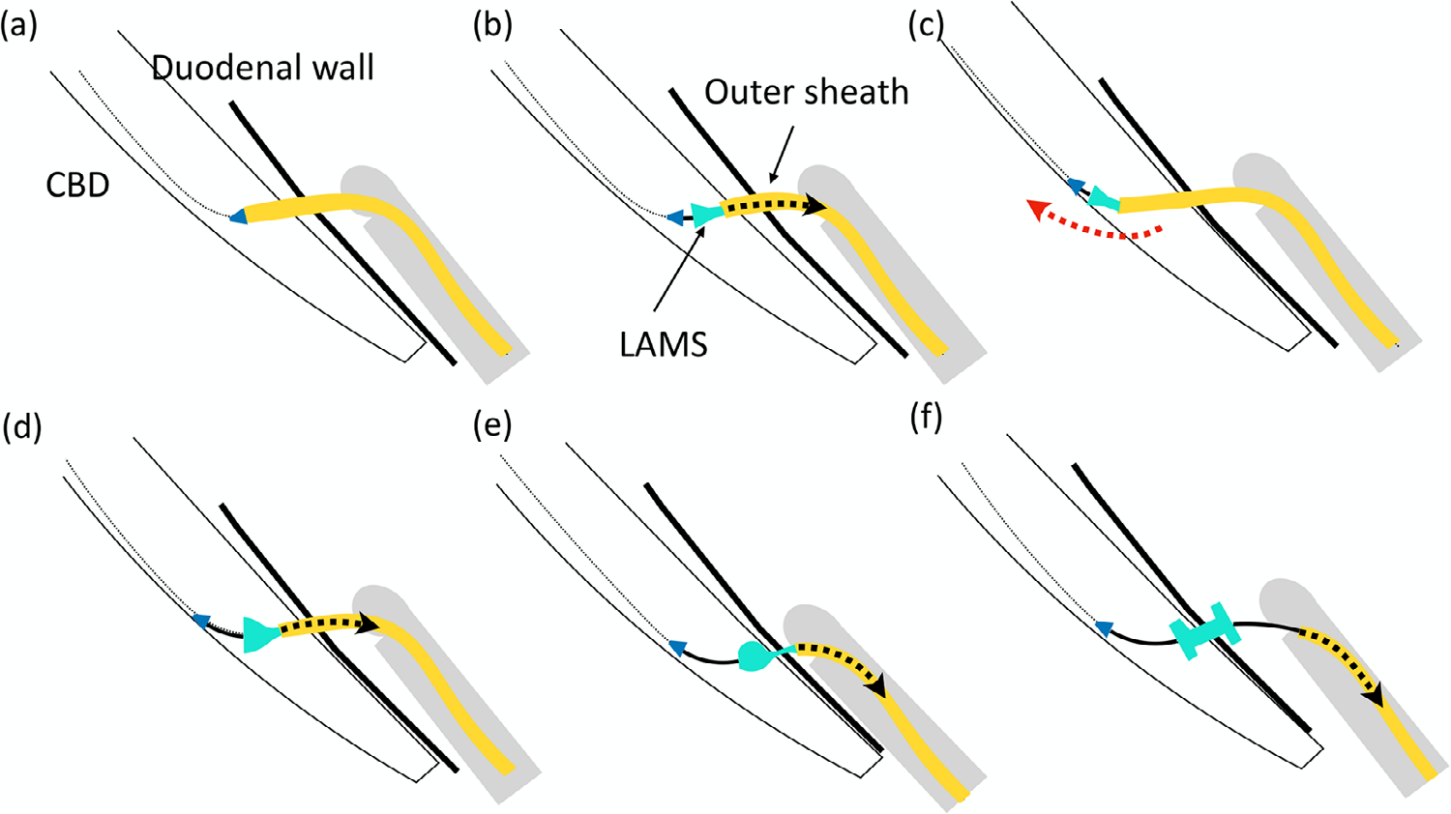
Push deployment technique for puncturing small bile duct in EUS‐CDS using LAMS. (a) Puncture the extrahepatic bile duct using the electrocautery tip of a lumen‐apposing metal stent and advance the preloaded guidewire toward the upstream bile duct. (b) Partially and slowly deploy the distal flange by withdrawing the outer sheath. Due to the inadequate runway, complete deployment of the stent may lead to the misplacement of the distal flange outside the bile duct. (c) Slowly advance the catheter further (as indicated by the dotted red arrow). (d) Deploy the distal flange again. If there is still insufficient runway for full deployment, repeat steps **9c** and **d** several times. (e) After confirming that the distal flange is fully opened within the bile duct, continue pulling the outer sheath using the intrascope channel technique. (f) Fully deploy the proximal flange in the digestive tract. EUS‐CDS, endoscopic ultrasound‐guided choledochoduodenostomy; LAMS, lumen‐apposing metal stents.

This technique is useful when targeting a small extrahepatic bile duct during EUS‐CDS using a LAMS to prevent malpositioning. The required size of the targeted organ, known as the “runway” distance, varies depending on the size of the LAMS. As the extrahepatic bile duct, which is the target of EUS‐CDS, is relatively small, securing an adequate runway distance can be challenging. In such cases, this technique is beneficial. Specifically, after puncturing the extrahepatic bile duct using the electrocautery tip of the LAMS and advancing the preloaded guidewire towards the liver, the distal flange is partially and slowly deployed. The catheter is then advanced further into the bile duct before continuing deployment. This maneuver may need to be repeated several times before the LAMS is fully deployed (Figure 9) [42].

### 3.11 Continuous Ascites Drainage (for all EUS‐BD)

Although there may be concerns regarding AE in patients with ascites who undergo EUS‐BD, studies on EUS‐CDS and HGS have not identified ascites as a significant factor associated with AE [16, 87]. However, EUS‐BD is unlikely to be performed in patients with massive ascites; thus, its safety in such cases remains unclear. One study demonstrated that continuous ascites drainage via a percutaneous drainage tube was effective in safely performing EUS‐HGS in patients with moderate or severe ascites [88]. This method, originally used in PTBD under similar circumstances, has been adopted for EUS and may be useful for ensuring a safe procedure in patients with massive ascites.

## 4 Conclusion

The use of EUS‐BD procedures is becoming increasingly widespread. Although it has generally been used as a rescue procedure when ERCP fails, it has already been considered a first‐line treatment in some situations. However, AEs can still occur, even with experts, and the recognition and management of these are critical to ensuring optimal patient care.

## Conflicts of Interest

The authors declare no conflicts of interest.
